# Tunable Valley Polarization and Valley Orbital Magnetic Moment Hall Effect in Honeycomb Systems with Broken Inversion Symmetry

**DOI:** 10.1038/srep13906

**Published:** 2015-09-11

**Authors:** Zhigang Song, Ruge Quhe, Shunquan Liu, Yan Li, Ji Feng, Yingchang Yang, Jing Lu, Jinbo Yang

**Affiliations:** 1 State Key Laboratory for Mesoscopic Physics and School of Physics, Peking University, Beijing 100871, China; 2 Collaborative Innovation Center of Quantum Matter, Beijing, China; 3 International Center for Quantum Materials, Peking University, Beijing, China; 4 State Key Laboratory of Information Photonics and Optical Communications, Beijing University of Posts and Telecommunications, Beijing 100876, China; 5 School of Science, Beijing University of Posts and Telecommunications, Beijing 100876, China

## Abstract

In this Letter, a tunable valley polarization is investigated for honeycomb systems with broken inversion symmetry such as transition-metal dichalcogenide MX_2_ (M = Mo, W; X = S, Se) monolayers through elliptical pumping. Compared to circular pumping, elliptical pumping is a more universal and effective method to create coherent valley polarization. When two valleys of MX_2_ monolayers are doped or polarized, a novel anomalous Hall effect (called valley orbital magnetic moment Hall effect) is predicted. Valley orbital magnetic moment Hall effect can generate an orbital magnetic moment current without the accompaniment of a charge current, which opens a new avenue for exploration of valleytronics and orbitronics. Valley orbital magnetic moment Hall effect is expected to overshadow spin Hall effect and is tunable under elliptical pumping.

Valleytronics has generated great interest^1^,^2^,^3^,^4^,^5^ in the field of condensed matter physics because it involves many novel phenomena such as valley Hall effect (namely, electrons belong to different valleys separate to opposite sides in the presence of an transverse electric field)^6^, valley-coupled spin Hall effect^7^, and valley magnetic moment (it stems from self-rotating of the wave packet and has opposite value at different valley)^8^. The valleys refer to the conduction-band minima (CBM) and valence-band maxima (VBM). In some materials, the valleys are located at inequivalent positions in the momentum space, leading to a novel degree of freedom. Manipulation of such a degree of freedom forms the basis of valleytronics. Transition-metal dichalcogenide MX_2_ (M = Mo, W; X = S, Se) monolayers are a perfect platform for valleytronics due to their intrinsic valley degree of freedom on honeycomb lattice and direct band gap in the range of visible light.

In order to realize 100% valley polarization, circularly polarized optical field is used to selectively excite either of the two valleys^9^,^10^,^11^. However, circularly polarized optical field cannot tune the valley polarization. If valley polarization is tunable, physical phenomena associated with the intermediate states between the unpolarized and fully polarized valley states can be observed, for examples, valley quantum coherence^12^ and some fractional-quantum-Hall states^13^. In the meantime, spin Hall effect from different valleys can be summed up, and thus valley-coupled spin Hall effect^14^ becomes more apparent in a partial valley-polarized state^7^.

A strong spin-orbital coupling (SOC) is required to generate valley-coupled spin Hall effect. However, SOC is not strong for most valleytronic materials, such as graphene, silicene and silicon. The Zeeman-type spin splittings in the VBM of MX_2_ monolayers are large enough (0.15–0.46 eV)^7^,^15^ to observe valley-coupled spin Hall effect with hole carriers. Unfortunately, the spin splittings in the CBM of MX_2_ monolayers are too small to observe the valley-coupled spin Hall effect with electron carriers. In analogy to spin Hall effect, there should be an orbital magnetic moment Hall effect^16^, which does not depend on SOC. Manipulation of orbital magnetic moment is the basis of orbitronics^17^. The valley-contrasting orbital magnetic moment (valley orbital magnetic moment) in MX_2_ monolayers consists of two parts: one is from the parent atomic orbitals, and the other is from lattice structure (valley magnetic moment). Remarkably, valley orbital magnetic moment is a few times larger than spin magnetic moment in MX_2_ monolayers. Atomic orbital magnetic moment Hall effect has been reported in *p*-doped Si bulk^16^,^17^, but valley-coupled orbital magnetic moment Hall effect, especially the part from lattice structure, has been rarely reported.

In this Letter, we have investigated the degree of polarization for two different valleys under elliptical pumping in MX_2_ monolayers. A novel Hall effect (called valley orbital magnetic moment Hall effect) driven by opposite Berry curvature of different valleys is proposed. Valley orbital magnetic moment Hall effect is tunable by elliptical pumping. Hence, a new avenue is opened to the exploration of valleytronics and orbitronics.

## Results and Discussion

In a honeycomb system with *C*_3h_ and particle-hole symmetry, to the first order in *k*, the two-band ***k·p*** Hamiltonian near the massive Dirac cone (around the *Κ* or *K*′ point) without including SOC can be described as follows^7^:


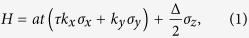


where *σ*_*α*_ denotes the Pauli matrices for the two basis functions, *t* represents the effective hopping integral, *a* is the lattice constant, *τ* = ± is the valley index, and Δ is the band gap. The pseudospin *τ* is a good quantum number near the *Κ* (*K*′) point, while it is not a good one away from the *Κ* (*K*′) point. All of the above parameters can be obtained by fitting the first-principles band structures.

Different polarization states of a monochromatic light wave with electric field projecting in the *x-y* plane could yield time-dependent components *E*_*x*_and *E*_*y*_ in cosine form as follows: 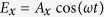 and 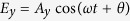, where *θ* is the phase retardation between *E*_x_ and *E*_y_, and the amplitude ratio is defined as 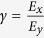. The coupling strength with optical fields of elliptical polarization is given by


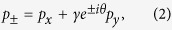


where *p*_*α*_ is the matrix element between the valence and conduction bands of the canonical momentum. *p*_*α*_ is given by


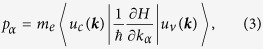


where 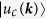 and 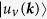 are the periodic parts of the conduction and valence bands of Bloch function, respectively, which are obtained by diagonalizing the matrix in Eq. 1, and *m*_e_ is the free electron mass. It is straightforward to derive an explicit form of *p*_±_ from Eqs 1. The ***k***-resolved degree of elliptical polarization is defined as


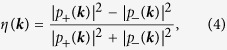


where *η*(***k***) is normalized by total absorption. According to Eqs 1, *η*(***k***) can be expressed as


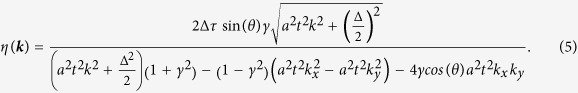


Then, the degree of elliptical polarization near the *Κ* (*K*′) point is given by


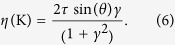


The term 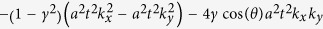 in Eq. 5 indicates that *η*(***k***) is anisotropic. However, this term is a second-order small one and negligible in the vicinity of the *K* (*K*′) point.

**Figure 1:**
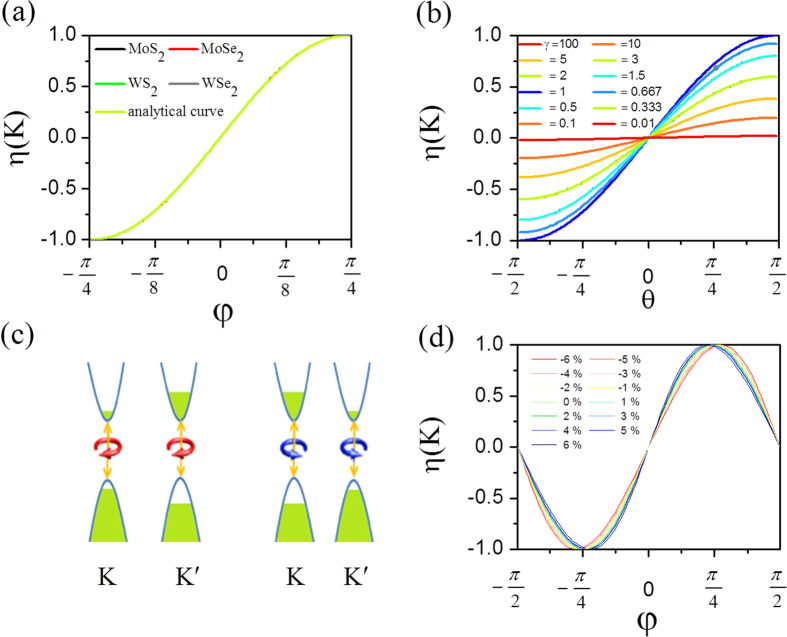
(**a**) Degree of elliptical polarization at the *K* point *η*(*K*) as a function of the phase retardation *θ* between the two components of electric field and the amplitude ratio *γ* calculated from Eq. 6 and the DFT method in MX_2_ monolayers. The DFT results of the four MX_2_ monolayers coincide with each other and are also in good agreement with Eq. 6. (**b**) *η*(*K*) as a function of rotation angle of the quarter wave plate *φ*. SOC has no influence on valley polarization around the *Κ* (*K*′) point. (**c**) Diagrammatic sketch showing that coherent valley polarization is created by right-(left-)hand elliptical pumping. (**d**) *η*(*K*) of WSe_2_ monolayers under different uniaxial stresses or strains as a function of *φ* calculated from the DFT method.

According to Eq. 6, the value of *η*(*K*) can continuously vary from +1 to −1 as the optical helicity of the excitation light changes. The helicity of the incident light can be continuously tuned by both changing *θ* (using electronically-controlled-liquid crystal retarders^18^) or adjusting the angle between polarization direction of the incident light (linearly polarized) and the fast axis of the modulator angle (*φ*) (see Fig. 1(a)). It is obvious that *γ* = tan(*φ*), and *θ* is 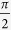, when a quarter-wave modulation is used. Thus, *η*(*K*) = sin(2*φ*) (Fig. 1(a)), and Eq. 6 is established with the condition that Δ > 0, namely, inversion symmetry is broken and the group of the wave vector at the band edges (the *K* and *K*′ points) is *C*_3_.

In order to further investigate the mechanism of the valley polarization, we performed density functional theory (DFT) simulations of *η*(*K*) as a function of *θ* and *γ*. In the calculation, we only consider the direct optical excitation with Δ***k*** = 0. There is no difference in the results of *η*(*K*) with and without SOC. Figure 1(a) plots the DFT calculated values of *η*(*K*) as a function of *φ* for MX_2_ monolayers, and (b) plots the DFT calculated values of *η*(*K*) as a function of *γ* and *θ* for MX_2_ monolayers. The DFT results of the four MX_2_ monolayers coincide with each other and are also in good agreement with the analytical ones. The elliptical pumping is illustrated in Fig. 1(c).

**Figure 2:**
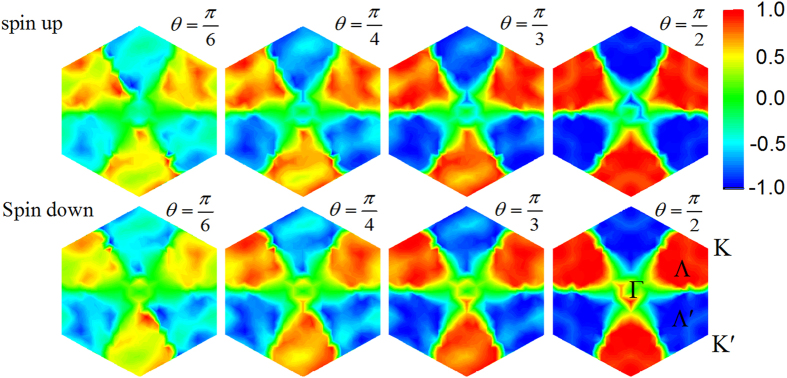
Spin-resolved degree of elliptical polarization *η*(***k***) in irreducible Brillouin zone for WSe_2_ monolayer with the phase retardation between the two components of electric field with *θ* = π/6, π/4, π/3, and π/2, respectively, calculated by the DFT method. The magnitude ratio is *γ* = 1. In cases of values of *θ* smaller than π/2, the lack of the *C*_3_ symmetry arises from the anisotropy of the elliptically polarized light.

As an example, the spin-resolved degrees of elliptical polarization *η*(***k***) for WSe_2_ monolayer in the irreducible Brillouin zone (BZ) with four different phase retardation are shown in Fig. 2. Our DFT calculation implies that the probability of the spin-flipped inter-band transitions is 3 orders of magnitude smaller than that of spin-conservation inter-band transitions. Thus the spin-flipped inter-band transitions can be neglected. We find that 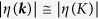 in a large region around the *K* (*K*′) point, because both the VBM and CBM and their wide range of adjacent region (Corresponding to the six pieces of blue or red arrow-shaped regions in Fig. 2 consist of mainly *d*-orbitals of the M atoms. The BZ can be divided into two parts, one around the *K* (*K*′) point, and the other around the Γ point. The former is spin-independent, where spin splitting is Zeeman-type. The later part is spin-dependent, where spin spliting is Rashba-type associated with the nearest-neighbor hopping. We find that the *η*(***k***) around the Γ point is spiral, determined by the Rashba-type spin texture^19^. It is interesting to find that 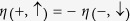 for the ***k*** far from the Γ point, where *τ* = ± is a good quantum number; while 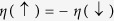 for the ***k*** near the Γ point, where *τ* is not a good quantum number any more.

When an in-plane uniaxial stress or strain is applied, the *C*_3_ symmetry of MX_2_ monolayer is broken. The Valley-selective elliptical dichroism replaces the circular dichroism. A right-hand (left-hand) elliptically polarized photon could be selectively absorbed around the *K* (*K*′) point, while a left-hand (right-hand) one was completely prohibited. This conclusion is similar to that of the recent experiment in which an electric field is used to break the *C*_3_ symmetry of the WSe_2_ monolayer^2^. The calculated elliptical polarization *η*(*K*) under different stresses or strains are shown in Fig. 1(d). It is obvious that the maximums of *η*(*K*) deviate from 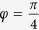. This indicates that an elliptically polarized light is more universe than a circularly polarized light in order to create complete valley polarization. Therefore, elliptical dichroism could be used to detect the planar stress or strain in MX_2_ monolayers.

**Figure 3:**
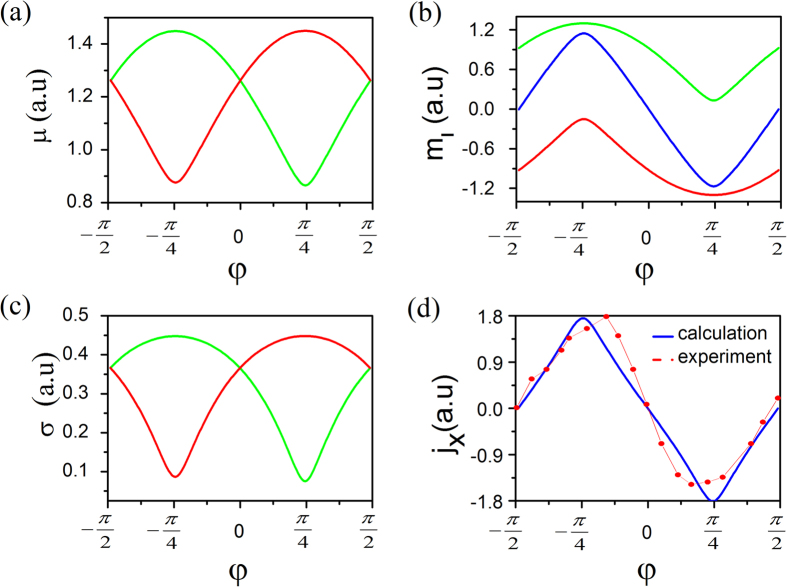
(**a**) Local chemical potential, (**b**) valley magnetization, (**c**) valley Hall conductivity, and (**d**) valley charge current in MX_2_ monolayers as a function of the rotation angle of the quarter wave plate *φ*. The red curve corresponds to the *K* point, green to the *K*′ point, and blue to the total. All these data are calculated from the analytic method. The experimental valley Hall current^1^ after transformation (see Supplementary Information) as a function of *φ* is given as a comparison. In our calculation, we adapt suitable I, *α* and *T* in Eq. 7 to make sure that the calculated amplitude of the Hall current equals to the measured value.

In order to understand the relationship among valley magnetization, valley orbital magnetic moment Hall effect, valley charge Hall current, and the degree of elliptical polarization, we proceed to derive analytic expression of local chemical potential *μ*, (see more details in Supplementary Information)


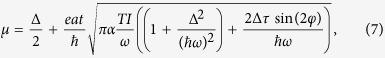


where *I* is the intensity of the incident light, *α* is the probability of absorption of one photon, and *T* is the average illumination time. Local chemical potential *μ* as a function of the amplitude ratio *γ* is plotted in Fig. 3(a).

**Figure 4:**
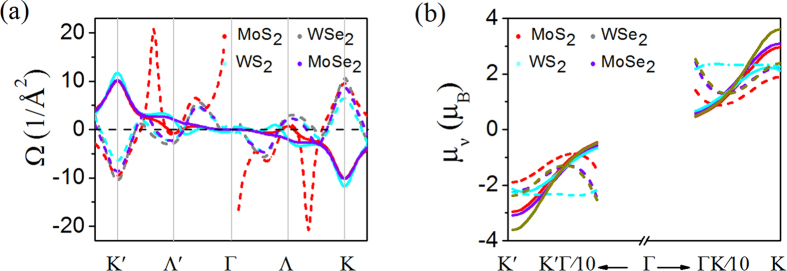
(**a**) Berry curvature *Ω*(***k***) and (**b**) valley magnetic moment *μ*(***k***) of MX_2_ monolayers calculated from the DFT method without including SOC. The solid and dashed curves represent values of the valence and conduction bands, respectively. The Berry curvatures of the conduction bands are cut off near the Γ point, where the values of Berry curvature are exceptionally large as a result of the band degeneracy.

According to particle-hole symmetry without including SOC, valley magnetizations of the valence and conduction bands are similar. The valley magnetization contributed from the lattice structure in the conduction band can be described by the Berry curvature as follows^6^,^20^:


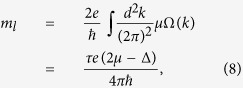


where *Ω* is Berry curvature and shown in Fig. 4(a). The integration is over all states below *μ*. The valley magnetism *m*_*l*_ is measurable. The orbital magnetization of *m*_*l*_ as a function of *φ* is plotted in Fig. 3(b), and the maximum peaks are at 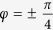.

The valley-contrasting magnetic moment originated from the lattice structure has the format of 
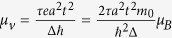
 in the low power limit 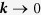. *μ*_*v*_ is in close analogy to spin except that the isotropic part of the effective mass in *μ*_*v*_ takes place of the free electron mass *m*_0_ in Bohr magneton^6^. The valley magnetic moments of CBM and VBM are in same direction, but in the two different valleys, the directions of the valley magnetic moments are opposite (see Fig. 4). Around the *Κ* (*K*′) point of MX_2_ monolayers, the particle-hole symmetry is totally preserved, and thus Berry curvatures of CBM and VBM are not absolutely opposite, leading to a result that the valley magnetic moments of CBM and VBM are inequivalent. Values of 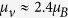 for the CBM and 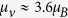 for the VBM are obtained for WSe_2_ monolayer.

In addition to the lattice structure contribution, the parent atomic orbital magnetic moment also contributes to the orbital magnetic moment. The CBM is mainly composed of 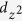-orbitals with *m* = 0, while the VBM is mainly of 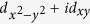-orbitals with an atomic orbital magnetic moment of 2*μ*_*B*_ around the *K* point and 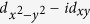-orbitals with an atomic orbital magnetic moment of −2*μ*_*B*_ around the *K*′ point^11^. The atomic orbital magnetic moment is also approximately twice of the spin moment, and its direction is the same as that of the valley magnetic moment^21^. Therefore, the valley orbital magnetic moment of the CBM is approximately 2.4 times of the spin magnetic moment, and that of the VBM is 5.6 times of the spin magnetic moment in the monolayer WSe_2_. The valley orbital magnetic moments of the conduction (valence) bands of two different spins are parallel.

**Figure 5:**
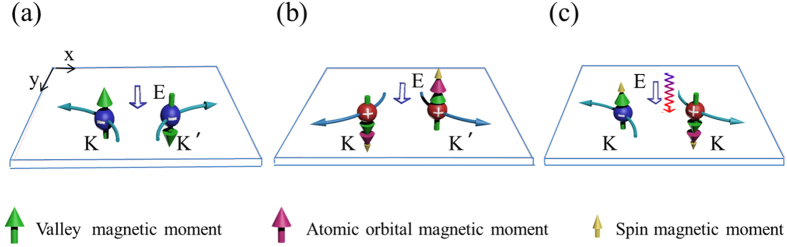
Valley orbital magnetic moment Hall effect in MX_2_ monolayers: (**a**) hole doping, (**b**) electron doping, and (**c**) electrons and holes excited by circularly polarized optical field. The blue spheres are electrons, while red ones are holes. Light blue arrows indicate movement of electrons or holes. Empty arrows indicate directions of electric fields. The helical line is light beam with left or right charity.

It was well established that an electron will acquire an anomalous transverse velocity when an in-plane electric field is applied^6^, which is proportional to the Berry curvature. Thus the carriers from different valleys (around the *K* and *K*′ points) carry opposite valley orbital magnetic moments and flow to the opposite transverse edges in the presence of an in-plane electric field. This will lead to an anomalous valley orbital magnetic moment Hall effect in close analogy to valley-coupled spin Hall effect. Valley orbital magnetic moment current (*J*) is defined as the average of the valley orbital magnetic moment per electron 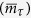 times the velocity operator 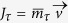, 
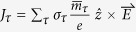
, where 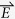 is electric field, 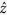 is the unit vector in the vertical direction, and *σ*_*τ*_ is the valley Hall conductivity given by^7^


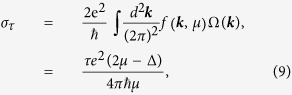


where the integration is over all states below *μ*. There are three kinds of valley orbital magnetic moment Hall effect: electron-doped, hole-doped and electron-hole pair (see Fig. 5). The former two are accompanied with no charge current. In the last case, *μ* is a function of *θ* and *φ*, and thus *σ*_*τ*_ can be adjusted by the helicity of incident light. If the edge states of samples are insulating, there is an accumulation of carriers with opposite orbital magnetic moment and Berry phase on opposite sides, which can be detected by Kerr or Faraday effects. If the edge states of samples are metallic, there may be one-dimensional channel for valley orbital magnetic moment. Although orbital magnetic moment current is not directly measurable, magnetic current can induce an electric field, which is an indirect signal to detect magnetic current. We should emphasize that valley orbital magnetic moment Hall effect is different from valley-coupled spin Hall effect in the origin of the magnetic moment, even though they both induce a magnetic current. With the improvement of technology, they may be distinguished in experiment not just in theory^22^.

If the average valley orbital magnetic moment 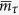 is replaced by electric charge in *J*_*τ*_, the common valley charge current is obtained:


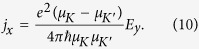


Similar to the valley Hall conductivity, the valley charge current is tunable by the helicity of incident light. Figure 3(c,d) show the valley Hall conductivity of the conduction bands (*σ*_*τ*_) and the valley charge current *j*_*x*_(*j* ∝ *J*) vs. angle *φ*, respectively. The dependence of valley charge current on *φ* shows a sine-shape format. Recently, valley charge Hall effect was successfully observed^1^. The observed period of the valley electric current on *φ* is in agreement with our theoretical results (see Fig. 3(d)).

MX_2_ monolayers are different from graphene mainly in two aspects. One is that inversion symmetry is explicitly broken in MX_2_ monolayers, and the other is that the band gaps of MX_2_ monolayers are in the visible frequency range^23^,^24^,^25^,^26^. Both of the two features are critical to realize valley orbital magnetic moment Hall effect. A large band gap corresponds to a long lifetime of electron-hole pairs and a broad distribution range of Berry curvature in momentum space. As a result, the number of the carriers carrying nonzero valley orbital magnetic moment is large, resulting in a high magnetization according to Eq. 8. In addition, since the two valleys in momentum space are far away, the inter-valley scattering can be strongly suppressed in MX_2_ monolayers^27^ and graphene^28^ (note that the inter-valley scattering here is supperessed but can still be observed^29^,^30^). The combination of a relatively long lifetime of electron-hole pairs and a suppressed inter-valley scattering induces a lifetime (about ten picoseconds) of the valley carriers long enough to detect valleytronics^29^,^31^,^32^,^33^. Actually, carriers with long lifetime and high density have been realized only in MX_2_ monolayers by circular pumping at the corners of the Brillouin zone^9^,^29^. In contrast, the band gap of graphene opened by inversion asymmetry is very small (<0.3 eV and in the infrared region)^34^,^35^, and Berry curvature is localized in the vicinity of the *K*(*K*′) point. Therefore, the carriers carrying nonzero valley orbital magnetic moment are few, resulting in a low magnetization value according to Eq. 8, although the valley magnetic moment is very large. Besides, the lifetime of electron-hole pairs at massive Dirac cones is very short in the graphene due to the small band gap. Taken together, it is difficult to realize and observe the valley orbital magnetic moment Hall effect in the graphene, while MX_2_ monolayers are perfect systems to realize and observe valley orbital magnetic moment Hall effect.

In summary, a concise analytical expression of *η*(*K*) related to the phase retardation *θ* between the two components of the electric field and the amplitude ratio *γ* is derived. It is found that elliptical pumping is a more general and effective method to create coherent valley polarization. A novel Hall effect driven by opposite Berry curvature at different valleys, namely, valley orbital magnetic moment Hall effect, is predicted and is expected to mask spin Hall effect in MX_2_ monolayers. The valley orbital magnetic moment Hall effect can generate orbital magnetic moment current without the accompaniment of a charge current. This valley orbital magnetic moment Hall effect can be tuned by elliptical pumping.

## Method

The calculation is performed with the VASP package within the framework of the projector augmented wave (PAW) pseudopotential method. The plane-wave basis set is used with a cutoff energy of 350 eV. The exchange-correlation functional is treated within Perdew-Burke-Ernzerh of generalized gradient approximation.

## Additional Information

**How to cite this article**: Song, Z. *et al.* Tunable Valley Polarization and Valley Orbital Magnetic Moment Hall Effect in Honeycomb Systems with Broken Inversion Symmetry. *Sci. Rep.* **5**, 13906; doi: 10.1038/srep13906 (2015).

## Supplementary Material

Supplementary Information
