# Supplementary material for: Tunable Valley Polarization and Valley Orbital Magnetic Moment Hall Effect in Honeycomb Systems with Broken Inversion Symmetry
Source: Sci Rep. 2015 Sep 11;5:13906. doi: 10.1038/srep13906 (PMC4566088; doi:10.1038/srep13906)
Supplement: Supplementary Information [file srep13906-s1.pdf]

Supplementary Information

Tunable Valley Polarization and Valley Orbital Magnetic Moment Hall Effect in Honeycomb Systems with Broken Inversion Symmetry

Zhigang Song[1], Ruge Quhe[1,4,5], Shunquan Liu[1], Yan Li [1,2], Ji Feng[2,3], YingchangYang[1], Jing Lu[1,2 *], Jinbo Yang[1,2*]

[1]State Key Laboratory for Mesoscopic Physics and School of Physics, Peking University, Beijing 100871, China

[2]Collaborative Innovation Center of Quantum Matter, Beijing, China

[3]International Center for Quantum Materials, Peking University, Beijing, China

[4]State Key Laboratory of Information Photonics and Optical Communications, Beijing University of Posts and Telecommunications, Beijing 100876, China

[5]School of Science, Beijing University of Posts and Telecommunications, Beijing

Corresponding author: jinglu@pku.edu.cn, jbyang@pku.edu.cn

Part 1

The photoluminescence power around the K (K') points using the low-energy Hamiltonian (1) is described by

$$W=\frac{4\pi}{\hbar}(\frac{e}{m_e})^2\frac{A^2\hbar\omega}{(\gamma^2+1)}\int|p_\pm|^2\delta[\varepsilon_c(k)-\varepsilon_v(k)-\hbar\omega]\frac{d^2k}{(2\pi)^2}$$

$$=\frac{A^2\omega^2e^2}{4\hbar}((1+\frac{\Delta^2}{(\hbar\omega)^2})+\frac{4\Delta\tau\gamma}{\hbar\omega(\gamma^2+1)}), \qquad (1)$$

where $\vec{A}$ is the magnetic vector potential of the linearly polarized light used to make elliptically polarized one. During an interval T, the number of the particle excited by elliptical photons can be described as:

$$n_e = T\alpha \frac{A^2 \omega e^2}{4\hbar^2}((1+\frac{\Delta^2}{(\hbar\omega)^2}) + \frac{4\tau\Delta\gamma}{\hbar\omega(\gamma^2+1)}) \quad , \tag{2}$$

where α is the probability that a photon is absorbed . According to Fermi-Dirac statistics,

$$n_e = \frac{1}{\pi a^2 t^2} \int_{\frac{\Delta}{2}}^{\infty} \frac{\varepsilon - \frac{\Delta}{2}}{e^{\beta(\varepsilon-\mu)}+1} d\varepsilon \quad , \tag{3}$$

where μ is the local chemical potential near the K and K' points. In the case of degenerate limit or low temperature limit ($\beta\mu > 3$), one obtains

$$n_e = \frac{(\mu - \frac{\Delta}{2})^2}{2\pi a^2 t^2}, \tag{4}$$

$$\mu = \frac{\Delta}{2} + \frac{eat}{2\hbar}\sqrt{2T\alpha A^2 \omega\pi((1+\frac{\Delta^2}{(\hbar\omega)^2}) + \frac{4\Delta\tau\gamma}{\hbar\omega(\gamma^2+1)})}$$

$$= \frac{\Delta}{2} + \frac{eat}{\hbar}\sqrt{\pi\alpha \frac{TI}{\omega}(1+\frac{\Delta^2}{\hbar^2\omega^2} + \frac{2\Delta\tau\sin(2\varphi)}{\hbar\omega})} \quad , \tag{5}$$

where $I = \frac{1}{2}A^2\omega^2$

Part 2

In Fig. 2b of Ref. 1 the Hall resistance is defined as $R_H = \frac{V_y}{I_x} = \frac{R_y I_y}{I_x}$, where I_x is longitudinal current, and R_y is a constant with resistance dimension. I_x is large and stable compared with I_y, so I_x can be considered as a constant. Hence $R_H \propto I_y$, which can be used to compare with our results.
